# Development of a rapid detection method for the macrolide resistance gene in *Mycobacterium avium* using the amplification refractory mutation system–loop-mediated isothermal amplification method

**DOI:** 10.1128/spectrum.02339-23

**Published:** 2024-02-16

**Authors:** Takayuki Inagaki, Shoki Asahi, Kenji Ogawa, Taku Nakagawa, Teruko Ohkura, Yukari Osada, Toshiaki Nikai, Kiyofumi Yamada, Tetsuya Yagi, Kei-ichi Uchiya

**Affiliations:** 1 Division of Pharmaceutical Sciences I, Faculty of Pharmacy, Meijo University, Nagoya, Aichi, Japan; 2 Department of Hospital Pharmacy, Nagoya University Hospital, Nagoya, Aichi, Japan; 3 Department of Hospital Pharmacy, Japan Organization of Occupational Health and Safety, Chubu Rosai Hospital, Nagoya, Aichi, Japan; 4 Department of Respiratory Medicine, National Hospital Organization, Higashinagoya National Hospital, Nagoya, Aichi, Japan; 5 Department of Medical Technique, Nagoya University Hospital, Nagoya, Aichi, Japan; 6 Department of Microbiology, Faculty of Pharmacy, Meijo University, Nagoya, Aichi, Japan; 7 Department of Neuropsychopharmacology and Hospital Pharmacy, Nagoya University Graduate School of Medicine, Nagoya, Aichi, Japan; 8 Department of Infectious Diseases, Nagoya University Graduate School of Medicine, Nagoya, Aichi, Japan; University of Cincinnati, Cincinnati, Ohio, USA

**Keywords:** 23S rRNA, amplification refractory mutation system, loop-mediated isothermal amplification, clarithromycin, drug-resistance gene, *Mycobacterium avium*

## Abstract

Macrolide antibiotics such as clarithromycin (CLR) and azithromycin are the key drugs used in multidrug therapy for *Mycobacterium avium* complex (MAC) diseases. For these antibacterial drugs, drug susceptibility has been correlated with clinical response in MAC diseases. We have previously demonstrated the correlation between drug susceptibility and mutations in the 23S rRNA gene, which confers resistance to macrolides. Herein, we developed a rapid detection method using the amplification refractory mutation system (ARMS)–loop-mediated isothermal amplification (LAMP) technique to identify mutations in the 23S rRNA gene of *M. avium*. We examined the applicability of the ARMS–LAMP method to genomic DNA extracted from six genotypes of *M. avium* clinical isolates. The *M. avium* isolates were classified into 21 CLR-resistant and 9 CLR-susceptible strains based on the results of drug susceptibility tests; the 23S rRNA genes of these strains were sequenced and analyzed using the ARMS–LAMP method. Sequence analysis revealed that the 9 CLR-sensitive strains were wild-type strains, whereas the 21 CLR-resistant strains comprised 20 mutant-type strains and one wild-type strain. Using ARMS–LAMP, no amplification from genomic DNAs of the 10 wild-type strains was observed using the mutant-type mismatch primer sets (MTPSs); however, amplification from the 20 mutant-type strain DNAs was observed using the MTPSs. The rapid detection method developed by us integrates ARMS–LAMP with a real-time turbidimeter, which can help determine drug resistance in a few hours. In conclusion, ARMS–LAMP might be a new clinically beneficial technology for rapid detection of mutations.

**IMPORTANCE:**

Multidrug therapy for pulmonary *Mycobacterium avium* complex disease is centered on the macrolide antibiotics clarithromycin and azithromycin, and resistance to macrolides is an important prognosticator for clinical aggravation. Therefore, it is important to develop a quick and easy method for detecting resistance to macrolides. Drug resistance is known to be correlated with mutations in macrolide resistance genes. We developed a rapid detection method using amplification refractory mutation system (ARMS)–loop-mediated isothermal amplification (LAMP) to identify a mutation in the 23S rRNA gene, which is a macrolide resistance gene. Furthermore, we examined the applicability of this method using *M. avium* clinical isolates. The rapid method developed by us for detection of the macrolide resistance gene by integrating ARMS–LAMP and a real-time turbidimeter can help in detection of drug resistance within a few hours. Since this method does not require expensive equipment or special techniques and shows high analytical speed, it would be very useful in clinical practice.

## INTRODUCTION

Pulmonary *Mycobacterium avium* complex (MAC) disease is an intractable infection caused by nontuberculous mycobacteria (NTM) and has no reliable therapeutic agent; patients may die due to respiratory failure (1–3). Recently, the morbidity of diseases caused by NTM, including MAC, has increased rapidly and already exceeds tuberculosis morbidity in Japan (4). Pulmonary MAC disease is treated with multidrug therapy, which is centered on the macrolide antibiotics clarithromycin (CLR) and azithromycin (AZM) (5). Resistance to macrolides is an important prognosticator for clinical aggravation. Therefore, it is important to devise a quick and easy method for detecting resistance to macrolides. The drug susceptibility for ethambutol, rifampin, and rifabutin, in the commonly used antimycobacterial regimen for MAC disease, has shown a poor correlation with clinical response (6). On the contrary, macrolides are the only antimicrobial agents for which the correlation between MAC *in vitro* drug susceptibility testing and clinical response has been demonstrated in controlled clinical trials (5, 6). However, problems in drug susceptibility testing include the requirement of a long growth time before the determination of results and the need for technical expertise. Therefore, to rapidly determine the presence or absence of drug resistance in MAC, we require a method to detect a macrolide resistance gene mutation. Previous studies have shown that more than 95% of macrolide-resistant MAC isolates have a point mutation in the 23S rRNA domain V (6). In most CLR-resistant strains, a point mutation is present in the macrolide binding site at position 2058 or 2059 (encoding adenine) of the peptidyl transferase-harboring active center in the domain V region of 23S rRNA (7). With a conventional investigation, we ascertained the correlation between drug susceptibility and drug resistance gene mutations (8). Furthermore, we applied the amplification refractory mutation system (ARMS)–PCR method, which is used to detect gene mutations, to develop a method for rapid detection of macrolide resistance genes (8). The ARMS–PCR-based macrolide resistance gene detection method developed by us enables determination of the presence of drug resistance in approximately 1 day compared to the conventional drug susceptibility tests, using which the results are usually obtained in more than 2 weeks. The ARMS method is designed to the primers that overlap the mutation site at the amplification origin of the target region. For the ARMS–PCR method, primers that overlapped with the mutation site at the 3′-end of the gene were designed. In addition to the mismatch at the 3′-end of the primer, another mismatched base was introduced at a site just upstream of the mutation site, thereby preventing amplification by the PCR, which makes annealing of the primers impossible.

However, the ARMS–PCR method requires technical expertise and specialized equipment, similar to that required for the conventional PCR-based method. In the present study, we focused on the loop-mediated isothermal amplification (LAMP) method. Using the LAMP method, the gene can be amplified under isothermal conditions, and thus, expensive devices or instruments are not required for this method (9). The reaction employs two sets of primers, outer primers (F3 and B3) and inner primers [forward inner primer (FIP) and backward inner primer (BIP)], that recognize six distinct regions in the target DNA. Amplification is mediated by a loop structure and relies on an auto-cycling procedure performed with strand displacement of *Bst* DNA polymerase under isothermal conditions. We propose the following method of amplification: four types of primers amplify six regions, including the target gene sequence, which is specifically amplified; amplification occurs at a constant temperature and does not require expensive equipment or instruments such as thermal cyclers that are used for the PCR. This method of amplification is highly efficient and can be performed with a very small amount of DNA in a short time. Moreover, this method allows visual detection of DNA, and agarose gel electrophoresis is not required for this method. Considering these properties, the LAMP method is expected to become a routinely used clinical diagnostic technique.

In the present study, we developed a method for rapid detection of macrolide resistance in *M. avium* using the ARMS–LAMP technique. We also analyzed the applicability of this method using clinical isolates of *M. avium*.

## RESULTS

### Clarithromycin-susceptibility testing and analysis of the DNA sequence corresponding to domain V of 23S rRNA

In this study, we evaluated the applicability of the ARMS–LAMP method to genomic DNA extracted from six genotypes of *M. avium* clinical isolates. Table 1 shows the results of the CLR-susceptibility tests performed using the BrothMIC NTM method on 30 clinical isolates. Twenty-one CLR-resistant strains of *M. avium* isolated from patients with pulmonary MAC disease exhibited MICs ≥32 mg/L. The remaining nine CLR-susceptible strains of *M. avium* isolated from patients with pulmonary MAC disease exhibited MICs ≤8 mg/L.

**TABLE 1:** MICs of clarithromycin, mutations of nucleotides at positions 2058 and 2059 in the domain V region of the 23S rRNA gene, and results of amplification refractory mutation system (ARMS)–loop-mediated isothermal amplification (LAMP) performed using *Mycobacterium avium* isolates^*d*^

Strain	Total number	MIC (µg/mL)	Genotype	ARMS–LAMP					
				WTPS	CA-MTPS	GA-MTPS	TA-MTPS	AC-MTPS	AG-MTPS
Reference strain									
*M. avium* 104	1	0.25	AA	+	–	–	–	–	–
*M. avium* ATCC 25291	1	0.25	AA	+	–	–	–	–	–
Clinical isolates									
CLR-susceptible strains	9	<8	AA	+	–	–	–	–	–
CLR-resistant strains	1	>32	AA	+	–	–	–	–	–
	1	>32	CA	–	+	–	–	–	–
	1^*a*^	>32	CA	+	+	–	–	–	–
	1^*b*^	>32	CA	–	+	+	–	–	–
	5	>32	GA	–	–	+	–	–	–
	6	>32	TA	+	–	–	+	–	–
	3	>32	AC	–	–	–	–	+	–
	1^*c*^	>32	AC	–	+	–	–	+	–
	2	>32	AG	–	–	–	–	–	+

^*a*^ AV-27 strain.

^*b*^ AV-47 strain.

^*c*^ AV-124 strain.

^*d*^ AC-MTPS, A2059C mutant-type mismatch primer set; AG-MTPS, A2059G mutant-type mismatch primer set; ARMS–LAMP, amplification refractory mutation system–loop-mediated isothermal amplification; CA-MTPS, A2058C mutant-type mismatch primer set; CLR, clarithromycin; GA-MTPS, A2058G mutant-type mismatch primer set; TA-MTPS, A2058T mutant-type mismatch primer set; WTPS, wild-type mismatch primer set.

We then sequenced the regions corresponding to domain V of the 23S rRNA genes of the 9 CLR-susceptible and the 21 CLR-resistant strains. The amount of DNA was measured using NanoDrop, and the average quantity of DNA extracted from the 30 strains was 7.84 ng/L. As shown in Table 1, the nine susceptible strains were wild-type and harbored the sequences 2058A and 2059A. In contrast, 20 of the 21 resistant strains were of the mutant type; 14 strains had a mutation at position 2058 and 6 had a mutation at position 2059. One strain was found to be CLR-resistant but had no mutation at either of these positions.

### Amplification refractory mutation system (ARMS)–loop-mediated isothermal amplification (LAMP) analysis

Amplification of the target DNA in an ARMS–LAMP analysis is indicated by the rising slope of the curve that is obtained by plotting the real-time turbidimeter readings (Fig. S1). Furthermore, LAMP products show a ladder-like pattern on agarose gel electrophoresis (Fig. S2). The amplified samples that tested positive/negative on real-time turbidimetric analysis also showed positive/negative results, respectively, when analyzed by electrophoresis. The results of the ARMS–LAMP analysis of the *M. avium* isolates are shown in Table 1 and Table S1. In all the wild-type strains harboring the sequences 2058A and 2059A, the two reference strains, the nine susceptible strains, and one resistant strain, amplification was observed using the wild-type mismatch primer set (WTPS) but not with the mutant-type mismatch primer sets (MTPSs); these genotypes harbored the sequences A2058C (CA-MTPS), A2058G (GA-MTPS), A2058T (TA-MTPS), A2059C (AC-MTPS), and A2059G (AG-MTPS). In the analysis using the MTPSs, only 1 amplified product was observed in 11 of the 21 resistant strains.

Moreover, discrepancies were observed between the results of ARMS–LAMP and sequence analyses of nine mutant strains, namely, AV-27 (A2058C mutant-type strain), AV-47 (A2058C mutant-type strain), AV-124 (A2059C mutant-type strain), and all A2058T mutant-type strains. Sequence analysis showed that the strain AV-27 had an A → C mutation at position 2058, whereas ARMS–LAMP analysis of this strain showed amplification not only with CA-MTPS but also with WTPS. Similarly, sequence analysis revealed that the AV-47 strain had an A → C mutation at position 2058, whereas ARMS–LAMP analysis of this strain showed amplification not only with CA-MTPS but also with GA-MTPS. For the strain AV-124, sequence analysis showed an A → C mutation at position 2059, whereas ARMS–LAMP analysis of this strain showed amplification not only with AC-MTPS but also with CA-MTPS. Furthermore, sequence analysis revealed that the six A2058T mutant-type strains had an A → T mutation at position 2058, whereas ARMS–LAMP analysis of these strains showed amplification not only with TA-MTPS but also with WTPS.

### Subpopulation analysis of strains that showed discrepancies between results of DNA sequencing and ARMS–LAMP analyses

As mentioned above, since discrepancies were observed in the results of DNA sequencing analysis and ARMS–LAMP analysis of nine mutant strains, we analyzed subpopulations of these strains. The strain AV-27 was cultured in a liquid medium and was subsequently plated onto Middlebrook 7H11C agar. Three colonies were randomly selected from the resulting colonies and were again subjected to analysis. As given in Table 2, drug susceptibility test results revealed that these subpopulations were susceptible to CLR, and sequence analysis showed that these subpopulations had no mutations at positions 2058 and 2059. Furthermore, ARMS–LAMP analysis of these subpopulations showed amplification with WTPS but not with CA-MTPS. Similarly, subpopulation analysis of the AV-47 strain revealed that three subpopulations showed amplification with CA-MTPS and no amplification with GA-MTPS in the ARMS–LAMP analysis, and sequence analysis showed that these subpopulations had an A2058C mutation. ARMS–LAMP analysis of three subpopulations of the AV-124 strain showed amplification with AC-MTPS and no amplification with CA-MTPS, and sequence analysis showed that these subpopulations had an A2059C mutation. Furthermore, discrepancies between the results of the two analyses were also seen for the six A2058T mutant-type strains. ARMS–LAMP analysis of subpopulations of two of the A2058T mutant strains, AV-207 and AV-392, revealed that all subpopulations showed amplification with both WTPS and TA-MTPS, and sequence analysis revealed that these subpopulations had an A2058T mutation.

**TABLE 2:** Subpopulation analysis of the strains that showed discrepancies between the estimation of mutations of nucleotides at positions 2058 and 2059 in the domain V region of the 23S rRNA gene and results of ARMS–LAMP analysis^*a*^

Strain	Total number	MIC (µg/mL)	Genotype	ARMS–LAMP				
				WTPS	CA-MTPS	GA-MTPS	TA-MTPS	AC-MTPS
AV-27	3	<8	AA	+	–			
AV-47	3	>32	CA		+	–		
AV-124	3	>32	AC		–			+
AV-207	3	>32	TA	+			+	
AV-392	3	>32	TA	+			+	

^*a*^ AC-MTPS, A2059C mutant-type mismatch primer set; ARMS–LAMP, amplification refractory mutation system–loop-mediated isothermal amplification; CA-MTPS, A2058C mutant-type mismatch primer set; GA-MTPS, A2058G mutant-type mismatch primer set; TA-MTPS, A2058T mutant-type mismatch primer set; WTPS, wild-type mismatch primer set.

## DISCUSSION

In this study, we developed a system for rapid detection of macrolide resistance in *M. avium* clinical isolates. Our results showed that macrolide resistance could be detected more simply and rapidly using the ARMS–LAMP method than that using the conventional methods of drug susceptibility testing.

**Fig 1:**
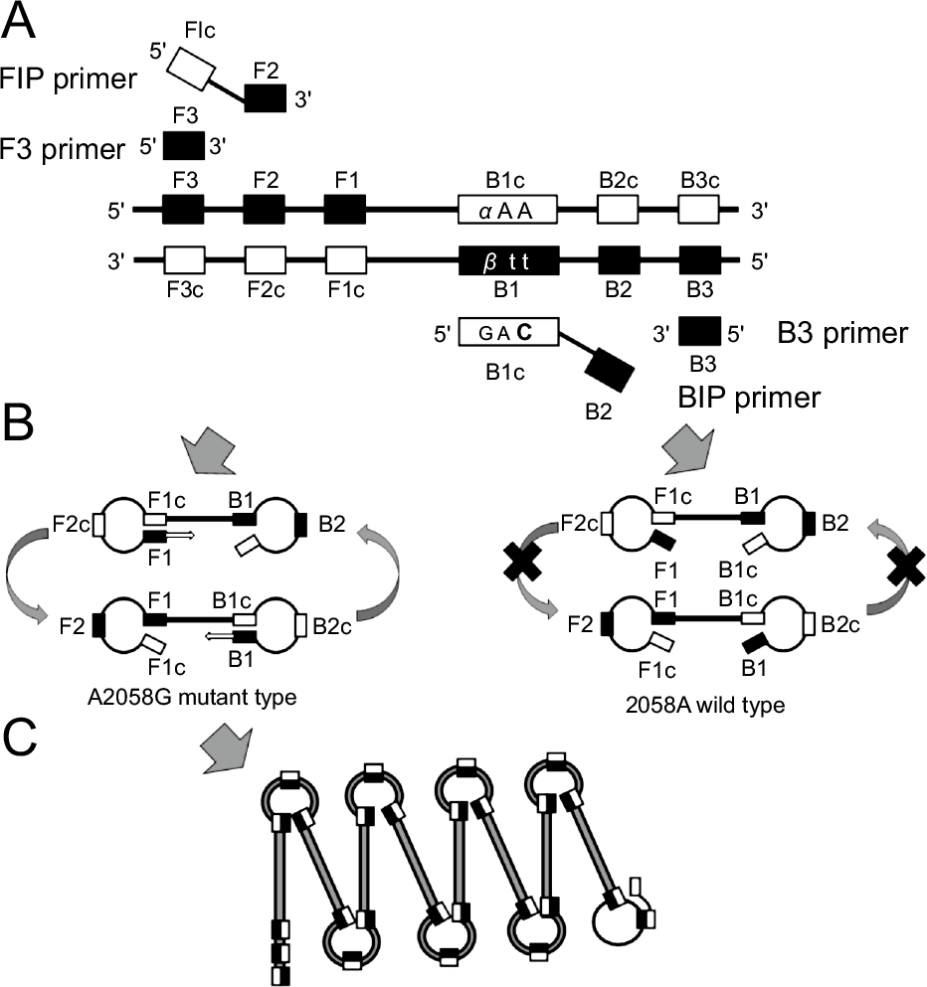
Design of A2058G mutant-type mismatch primers for amplification of the clarithromycin (CLR) resistance gene. These primers were used for the amplification refractory mutation system (ARMS)–loop-mediated isothermal amplification (LAMP) assay. (**A**) A strand-displacing DNA polymerase extends the DNA from the forward inner primer (FIP) while detaching the DNA chain. The outer primer F3 binds to its complementary region on the DNA strand to displace the newly synthesized DNA. An analogous reaction is performed by backward inner primer (BIP) and outer primer B3. α (α = A, wild type; G, A2058G) and β (β = T, wild type; C, A2058G) indicate the point mutations at position 2058 of the 23S rRNA gene. The bold base indicates the mismatched base C (cytosine). **(B**) The synthesized DNA strand self-anneals because of the complementary region at both ends and forms 'dumbbell-shaped' structures. **(C**) After repeated rounds, a complementary region on the same chain is amplified.

The ARMS–LAMP method is used for detection of point mutations in target genes as it helps in identifying mismatches using LAMP primers. As shown in Fig. 1, BIP primers harboring the B1c region are designed to overlap with the mutated site at the 5′-end. In addition to the mismatch at the 5′-end of the BIP primer, another mismatched base is introduced at a site just upstream of the mutated site, thereby preventing amplification by LAMP, which makes annealing of the primers impossible (10). Since mutations occur at two adjacent nucleotides, primers were designed so that these two sites were positioned at the 5′-end. Cytosine (C) was selected as the mismatched base because of the high level of mismatch strength that it shows with thymine (T) that is present in the template DNA (11–13). In this study, measurements were carried out using approximately 23 ng of DNA extracted from clinical isolates. Since the sensitivity and the specificity of the ARMS–LAMP method were high, the amount of DNA was also adequate. Tamura et al. reported that detection in the LAMP reaction is possible even with a small amount of DNA (up to 10 pg) and that approximately 10 pg of DNA can be extracted from a single sputum sample (14). In the future, we will examine whether this method is feasible for the assessment of *M. avium* that is present in sputum samples.

We detected the LAMP products by electrophoresis on an agarose gel and real-time turbidimetric analysis in the present study. It was found that all samples that tested positive/negative in the real-time turbidimetric analysis also showed positive/negative results, respectively, when they were analyzed by electrophoresis. The real-time turbidimetric inspection of LAMP products serves as an excellent detection method for clinical diagnosis. Previously, we developed a rapid assay based on the ARMS-PCR for detecting the 23S rRNA mutations at positions 2058–2059 in MAC (8). Although the ARMS–PCR method could detect the 23S rRNA mutations with high accuracy, it required the use of thermal cyclers and agarose gel electrophoresis. Besides, the whole genome sequence is useful when investigating unknown mutations or broader genetic variations, while the ARMS-LAMP method is useful when investigating specific known mutations. Additionally, the ARMS-LAMP method is cost-effective and expeditious, especially when the target sequence region is short. In contrast, the whole-genome sequencing workflow is more complex and time-consuming. In other words, it eliminates the need for gel electrophoresis and staining with ethidium bromide, making it more convenient and cost-effective compared to sequencing methods. A previous study reported the presence of turbidity in macroscopic samples (15). However, in our experiments, it was difficult to visually detect a change in turbidity. Bista et al. reported that the turbidimetric and colorimetric assessment of the LAMP reaction is subjective when determined by visual observation alone, as it relies on an individual’s perception of color (16). Since we are aiming for practical use of the LAMP method in general hospitals and practicing clinics, we will further analyze if LAMP products can be visually detected without using special equipment.

Although the ARMS–LAMP method has some advantages over other assays, it also has limitations in the detection of CLR-resistant *M. avium*. First, by comparing the results of drug susceptibility tests, sequence analysis, and ARMS–LAMP analysis, we confirmed that the CLR-susceptible strains had no mutations at positions 2058 and 2059 and yielded amplified products only with WTPS in the ARMS–LAMP analysis. In contrast, 1 out of the 21 CLR-resistant strains was determined to be wild-type based on the results of sequence and ARMS–LAMP analyses. The sequence analysis of 23S rRNA revealed that this strain was completely identical to the wild type. The potential reasons for the existence of this strain may be involvement of drug resistance mechanisms other than mutations in the 23S rRNA gene, such as an efflux pump, and mutations at other positions in the 23S rRNA gene or at other genomic sites (17). Among the 21 CLR-resistant strains, the strains AV-27, AV-47, and AV-124 showed discrepancies in the results of sequence and ARMS–LAMP analyses. These discrepancies in results may be because of the difficulties in isolation of the *M. avium* isolates, as mixtures of drug-susceptible and drug-resistant subpopulations in various proportions were observed in the subpopulation analysis. Indeed, our previous studies have demonstrated that three CLR-resistant *M. avium* strains had mixtures of drug-susceptible and drug-resistant subpopulations present in the samples (8). In the case of the A2058T mutant strains that showed discrepancies between results of the sequence analysis and the ARMS–LAMP analysis, the reason for the discrepancies might be nonspecific binding of the primers used in the ARMS–LAMP analysis, because for both sequence and ARMS–LAMP analyses, the results of the subpopulation analyses were similar to those obtained at the pre-isolation stage. Overall, the ARMS–LAMP analysis using MTPS showed no amplification from the wild-type strains and amplification from all mutant-type strains. Second, the 23S rRNA sequence of *M. intracellulare* is slightly different from that of *M. avium* (18). Therefore, we were unable to design a primer set corresponding to a clinical isolate of *M. intracellulare*, but we will design a primer set for MAC containing *M. intracellulare* in the future. Third, in this study, we used the ARMS–LAMP method to analyze a limited number of *M. avium* isolates. We were unable to assess the efficacy of the ARMS–LAMP method using the A2059T genotype because we were unable to isolate the A2059T mutant strains reported by Griffith et al. (19). The current ARMS–LAMP method alone cannot be used to identify all CLR-resistant strains because at least 5% of CLR-resistant strains have no mutations at positions 2058 and 2059 of the 23S rRNA gene (6, 20). Therefore, comprehensive examination for definitive judgment of resistant strains needs to be conducted by analyzing the clinical symptoms and the results of the drug susceptibility tests and the ARMS–LAMP analysis.

In conclusion, we developed a rapid method for detection of macrolide-resistant *M. avium* using ARMS–LAMP analysis with real-time turbidimetry that can help in determination of drug resistance in a few hours. Our results also showed the usefulness of this method in detection of *M. avium* clinical isolates. In the future, further studies are required to enable direct detection of macrolide-resistant *M. avium* in sputum from patients who receive long-term multi-drug combination therapy after they are diagnosed with diseases caused by *M. avium*.

## MATERIALS AND METHODS

### Strains

*M. avium* strain 104 and *M. avium* ATCC 25291 were used as the reference strains (21). The clinical isolates used in this study were provided by the Higashinagoya National Hospital of the National Hospital Organization in Aichi Prefecture, Japan. These clinical isolates consisted of 30 *M*. *avium* strains isolated from patients diagnosed with pulmonary MAC infection. Notably, all clinical isolates were collected from patients, regardless of their clinical backgrounds and previous use of antibacterial therapy. Furthermore, only one strain from each patient was analyzed in this study. All clinical isolates were determined to be *M. avium* using the Cobas TaqMan mycobacterium test (Roche Diagnostic Systems, Inc., Basel, Switzerland) (22). *M. avium* isolates were cultured at 37°C in 5 mL Middlebrook 7H9 liquid medium supplemented with 10% oleic acid/albumin/dextrose/catalase for 1–3 weeks, and subsequently, 5 mL of the culture medium was transferred to the Mycobroth liquid medium (Kyokuto Pharmaceuticals Industrial Co., Ltd., Tokyo, Japan).

### Drug susceptibility testing

BrothMIC NTM (Kyokuto Pharmaceutical Industrial Co., Ltd.) was used to determine the CLR susceptibility of the *M. avium* strains at pH 7.4, according to the manufacturer’s instructions (23). BrothMIC NTM is compliant with CLSI M24-A (using the Middlebrook 7H9 medium) (24). The test concentration ranges were 0.03 to 32 mg/L for CLR. Based on the criteria described in the BrothMIC NTM manual, strains with MICs ≤8 mg/L for CLR at pH 7.4 were considered susceptible to CLR, and those with MICs ≥32 mg/L at this pH were considered resistant to CLR. Similarly, according to compliance with CLSI M24 3rd ed and CLSI M24S 2nd ed (using Muller–Hinton medium), strains with MIC ≤8 mg/L were considered susceptible to CLR, and those with MICs ≥32 mg/L were considered resistant to CLR (6, 25). The effect of the medium on the macrolide susceptibility results of MAC is within an acceptable range (26).

### Analysis of the DNA sequence corresponding to domain V of 23S rRNA

DNA of the 21 CLR-resistant strains and the nine CLR-susceptible strains (as determined by the drug susceptibility test results) was extracted using the illustra bacteria genomicPrep Mini Spin Kit (GE Healthcare UK Ltd., Buckinghamshire, England), according to the manufacturer’s instructions. The quantity of extracted DNA was estimated using a NanoDrop spectrophotometer (Thermo Fisher Scientific, Tokyo, Japan). PCR was performed to amplify the region corresponding to domain V of the 23S rRNA gene, according to the method described by Jamal et al. (27). The PCR mixture (50 µL) was composed of 2 µL DNA solution, 1 U AmpliTaq Gold DNA polymerase (Applied Biosystems, Foster City, CA, USA), 5 µL of 2 mM deoxynucleoside triphosphate mixture, 5 µL of 10× PCR buffer, and 1 µL each of the primers (23SF I, 5′-TTTAAGCCCCAGTAAACGGC-3′; 23 SR III, 5′-GTCCAGGTTGAGGGAACCTT-3′) at 25 mM. The reactions were carried out using a GeneAmp 9700 PCR system (Applied Biosystems). The PCR conditions were as follows: 1 cycle at 95°C for 10 min; 35 cycles at 94°C for 1 min, 55°C for 1 min, and 72°C for 1 min; and 1 cycle at 72°C for 7 min. The PCR products were electrophoresed along with the TrackIt 50 bp DNA ladder (Invitrogen, San Diego, CA, USA) on 2% agarose gel (Invitrogen). The resulting PCR products were purified using a GenElute PCR Clean-Up kit (Sigma-Aldrich, St Louis, MO, USA), and sequence analysis was performed using the same primers as those used for the PCR. The resulting nucleotide sequences were compared with the genomic sequence data available for the *M. avium* strain 104 (GenBank accession number NC008595). Alignment of the nucleotide sequences was performed using CLC Genomics Workbench version 12.0.3 (QIAGEN NV, Hilden, Germany).

### Amplification refractory mutation system (ARMS)–loop-mediated isothermal amplification (LAMP) method

**Fig 2:**
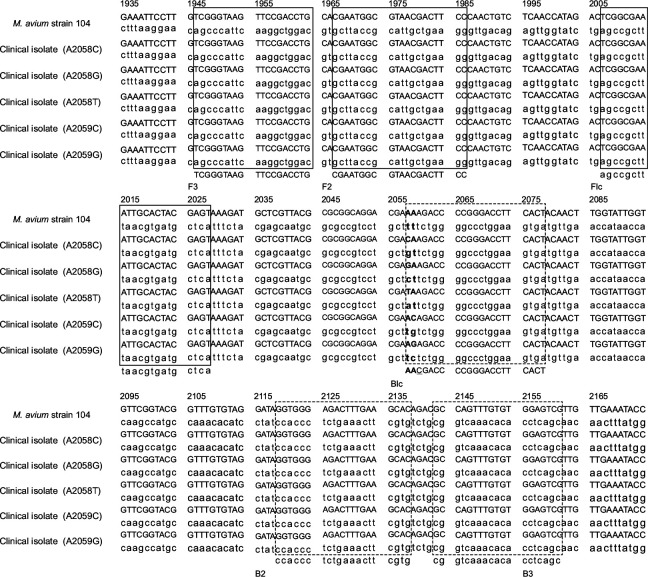
Alignment of the nucleotide sequences of the macrolide binding site at the domain V region of the 23S rRNA gene. The constructed LAMP primer sets are shown in solid boxes (forward primers, F1–3) and dashed boxes (backward primers, B1–3). Bold letters indicate the point mutations at positions 2058 and 2059 of the 23S rRNA gene.

DNA of the 21 CLR-resistant strains and the 9 CLR-susceptible strains (as determined by the drug susceptibility test results) was extracted using the illustra bacteria genomicPrep Mini Spin Kit (GE Healthcare UK Ltd.), according to the manufacturer’s instructions. Primers (armslampclr-F3, armslampclr-B3, armslampclr-FIP, armslampclr-BIPaa, armslampclr-BIPca, armslampclr-BIPga, armslampclr-BIPta, armslampclr-BIPac, and armslampclr-BIPga) were designed using PrimerExplorer version 5 software (https://primerexplorer.jp/) based on the nucleotide sequence of 23S rRNA of the *M. avium* strain 104 (GenBank accession number NC008595) (Table 3; Fig. 2). For the detection of wild-type strains with sequences 2058A and 2059A, common primers (armslampclr-F3, armslampclr-B3, and armslampclr-FIP) and armslampclr-BIPaa (WTPS) were used for amplification. For the detection of A2058G mutant-type strains, common primers and armslampclr-BIPga (GA-MTPS) were used (Fig. 1). For the detection of other mutations at positions A2058C, A2058T, A2059C, and A2059G, common primers, in addition to armslampclr-BIPca (CA-MTPS), armslampclr-BIPta (TA-MTPS), armslampclr-BIPac (AC-MTPS), and armslampclr-BIPag (AG-MTPS), respectively, were used.

**TABLE 3:** Primer sequences used for ARMS–LAMP analysis^*c*^^,^*^d^*^,^^*e*^

Primer	Sequence (5′ → 3′)	Length (bp)
armslampclr-F3	TCGGGTAAGTTCCGACCTG	19
armslampclr-B3	CGACTCCACACAAACTGGC	19
armslampclr-FIP^*a*^	ACTCGTAGTGCAATTTCGCCGA-CGAATGGCGTAACGACTTCC	42
armslampclr-BIPaa^*b*^	AA**C**GACCCCGGGACCTTCACT-GTGCTTCAAAGTCTCCCACC	41
armslampclr-BIPca^*b*^	CA**C**GACCCCGGGACCTTCACT-GTGCTTCAAAGTCTCCCACC	41
armslampclr-BIPga^*b*^	GA**C**GACCCCGGGACCTTCACT-GTGCTTCAAAGTCTCCCACC	41
armslampclr-BIPta^*b*^	TA**C**GACCCCGGGACCTTCACT-GTGCTTCAAAGTCTCCCACC	41
armslampclr-BIPac^*b*^	AC**C**GACCCCGGGACCTTCACT-GTGCTTCAAAGTCTCCCACC	41
armslampclr-BIPag^*b*^	AG**C**GACCCCGGGACCTTCACT-GTGCTTCAAAGTCTCCCACC	41

^*a*^ The FIP primer consists of F2 and the complementary strand of F1 (F1c).

^*b*^ The BIP primer consists of B2 and the complementary strand of B1 (B1c).

^*c*^ Underlined nucleotides show the location of each single-nucleotide polymorphism in 23S rRNA.

^*d*^ Nucleotides represented in bold letters show the locations of deliberate mismatches for ARMS.

^*e*^ bp, base pair; F3 and B3, outer primers; FIP and BIP, inner primers.

ARMS–LAMP reactions were performed using a Loopamp DNA Amplification Kit (Eiken Chemical, Tokyo, Japan). The reaction mixtures were prepared by adding the following reaction components to 3 µL of the DNA solution: 1 µL each of the inner primers (armslampclr-FIP and armslampclr-BIP) at 1.6 µM; 1 µL each of the outer primers (armslampclr-F3 and armslampclr-B3) at 0.2 µM; 1 µL of *Bst* DNA Polymerase (Eiken Chemical); 12.5 µL of the reaction mix; the final volume was made up to 25 µL using sterile distilled water. The ARMS–LAMP reaction mixtures were incubated at 63°C for 60 min and then heated at 80°C for 5 min to stop the reaction. Therefore, the total time required from DNA extraction to confirmation of reaction results is approximately 3 h or less. The Loopamp Control Set DNAs (Eiken Chemical) provided in the kit were used as negative and positive controls for the reaction. The LAMP reaction was evaluated using the Loopamp Real-time Turbidimeter (LoopampEXIA; Eiken Chemical Tokyo, Japan) that measures turbidity at 650 nm every 6 seconds and automatically plots the slope (Fig. S1). LAMP reactions were evaluated using a Loopamp real-time turbidity meter A positive reaction was defined as turbidity above a threshold value of 0.1 within 60 min of the beginning of the reaction. Furthermore, the LAMP products were electrophoresed on 2.0% agarose gel and stained with ethidium bromide to confirm the results of the reaction (Fig. S2).

### Subpopulation analysis of strains that showed discrepancies between results of DNA sequence and ARMS–LAMP analyses

Strains AV-27, AV-47, and AV-124, which showed a discrepancy between results of the 23S rRNA gene sequence analysis and the ARMS–LAMP analysis, and strains AV-207 and AV-392 of the A2058T mutant-type isolates, which showed amplification with WTPS and TA-MTPS, were further investigated by subpopulation analysis. The strains were cultured at 37°C for 1–3 weeks in 5 mL of Middlebrook 7H9 liquid medium supplemented with 10% oleic acid/albumin/dextrose/catalase and were plated onto Middlebrook 7H11C agar (Nippon Becton Dickinson Co., Ltd., Tokyo, Japan). Three of the colonies were randomly selected and cultured in Mycobroth liquid medium (Kyokuto Pharmaceuticals). The cultures were again subjected to drug susceptibility tests using BrothMIC NTM. The analysis of the DNA sequence corresponding to domain V of the 23S rRNA gene and ARMS–LAMP was conducted as described in the previous paragraphs.

## SUPPLEMENTAL MATERIAL

Supplemental material spectrum.02339-23-s0001.docx Table S1; Fig. S1 and S2.

ASM does not own the copyrights to Supplemental Material that may be linked to, or accessed through, an article. The authors have granted ASM a non-exclusive, world-wide license to publish the Supplemental Material files. Please contact the corresponding author directly for reuse.
