# Supplementary material for: Development of a rapid detection method for the macrolide resistance gene in Mycobacterium avium using the amplification refractory mutation system–loop-mediated isothermal amplification method
Source: Microbiol Spectr. 2024 Feb 16;12(4):e02339-23. doi: 10.1128/spectrum.02339-23 (PMC10986505; doi:10.1128/spectrum.02339-23)
Supplement: Supplemental material — Table S1; Fig. S1 and S2. [file spectrum.02339-23-s0001.docx]

**SUPPLEMENTAL MATERIAL**

**TABLES**

**Table S1.** Amount of DNA determined using NanoDrop; MICs of clarithromycin, mutations of nucleotides at positions 2058 and 2059 in the domain V region of the 23S rRNA gene, and results of amplification refractory mutation system (ARMS)–loop-mediated isothermal amplification (LAMP) performed using *Mycobacterium avium* isolates.

| Strain | NanoDrop | MIC | Genotype | ARMS–LAMP | | | | | |
| --- | --- | --- | --- | --- | --- | --- | --- | --- | --- |
|  | (ng/µL) | (µg/mL) |  | WTPS | CA-MTPS | GA-MTPS | TA-MTPS | AC-MTPS | AG-MTPS |
| Reference strain |  |  |  |  |  |  |  |  |  |
| *M. avium* 104 | 10.0 | 0.25 | AA | + | - | - | - | - | - |
| *M. avium* ATCC 25291 | 9.4 | 0.25 | AA | + | - | - | - | - | - |
| Clinical isolates |  |  |  |  |  |  |  |  |  |
| AV-1 | 18.2 | >32 | TA | + | - | - | + | - | - |
| AV-6 | 9.0 | >32 | AC | - | - | - | - | + | - |
| AV-9 | 9.4 | 0.25 | AA | + | - | - | - | - | - |
| AV-10 | 9.0 | 4 | AA | + | - | - | - | - | - |
| AV-11 | 14.7 | 0.25 | AA | + | - | - | - | - | - |
| AV-15 | 6.8 | 0.25 | AA | + | - | - | - | - | - |
| AV-22 | 22.3 | >32 | AC | - | - | - | - | + | - |
| AV-25 | 6.8 | >32 | GA | - | - | + | - | - | - |
| AV-26 | 3.3 | >32 | TA | + | - | - | + | - | - |
| AV-27 | 4.9 | >32 | CA | + | + | - | - | - | - |
| AV-44 | 6.4 | >32 | AC | - | - | - | - | + | - |
| AV-47 | 4.9 | >32 | CA | - | + | + | - | - | - |
| AV-55 | 22.3 | >32 | AA | + | - | - | - | - | - |
| AV-60 | 5.8 | >32 | GA | - | - | + | - | - | - |
| AV-65 | 14.1 | 0.25 | AA | + | - | - | - | - | - |
| AV-71 | 14.3 | >32 | GA | - | - | + | - | - | - |
| AV-82 | 17.2 | >32 | CA | - | + | - | - | - | - |
| AV-84 | 7.8 | 0.25 | AA | + | - | - | - | - | - |
| AV-88 | 23.8 | >32 | AG | - | - | - | - | - | + |
| AV-94 | 6.6 | >32 | GA | - | - | + | - | - | - |
| AV-111 | 14.7 | >32 | TA | + | - | - | + | - | - |
| AV-124 | 7.9 | >32 | AC | - | + | - | - | + | - |
| AV-136 | 5.5 | >32 | GA | - | - | + | - | - | - |
| AV-201 | 16.2 | 0.06 | AA | + | - | - | - | - | - |
| AV-207 | 22.6 | >32 | TA | + | - | - | + | - | - |
| AV-265 | 6.6 | 0.25 | AA | + | - | - | - | - | - |
| AV-308 | 16.8 | >32 | AG | - | - | - | - | - | + |
| AV-317 | 14.2 | >32 | TA | + | - | - | + | - | - |
| AV-392 | 22.6 | >32 | TA | + | - | - | + | - | - |
| AV-527 | 7.1 | 0.25 | AA | + | - | - | - | - | - |

AC-MTPS, A2059C mutant-type mismatch primer set; AG-MTPS, A2059G mutant-type mismatch primer set; ARMS–LAMP, amplification refractory mutation system–loop-mediated isothermal amplification; CA-MTPS, A2058C mutant-type mismatch primer set; CLR, clarithromycin; GA-MTPS, A2058G mutant-type mismatch primer set; TA-MTPS, A2058T mutant-type mismatch primer set; WTPS, wild-type mismatch primer set.

**Supplemental figure legends**

**Fig. S1.** A representative turbidity judgment graph generated using the A2058G mutant type mismatch primer set for amplification of the clarithromycin (CLR) resistance gene. Real-time sensitivity of amplification refractory mutation system (ARMS)–loop-mediated isothermal amplification (LAMP) was monitored by measuring turbidity (optical density at 650 nm). The threshold value was 0.1, and when turbidity was > 0.1, it was considered a positive reaction in the ARMS–LAMP analysis. AA: wild type; AC: A2059C mutant type; AG: A2059G mutant type; CA: A2058C mutant type; GA: A2058G mutant type; NC: negative control; PC: positive control; TA: A2058T mutant type.

**Fig. S2.** The products of ARMS–LAMP obtained using A2058G mutant type mismatch primer set for amplification of the CLR resistance gene. A ladder-like pattern observed on 2.0 % agarose gel electrophoresis was considered as positive amplification. AA: wild type; AC: A2059C mutant type; AG: A2059G mutant type; CA: A2058C mutant type; GA: A2058G mutant type; NC: negative control; PC: positive control; TA: A2058T mutant type.


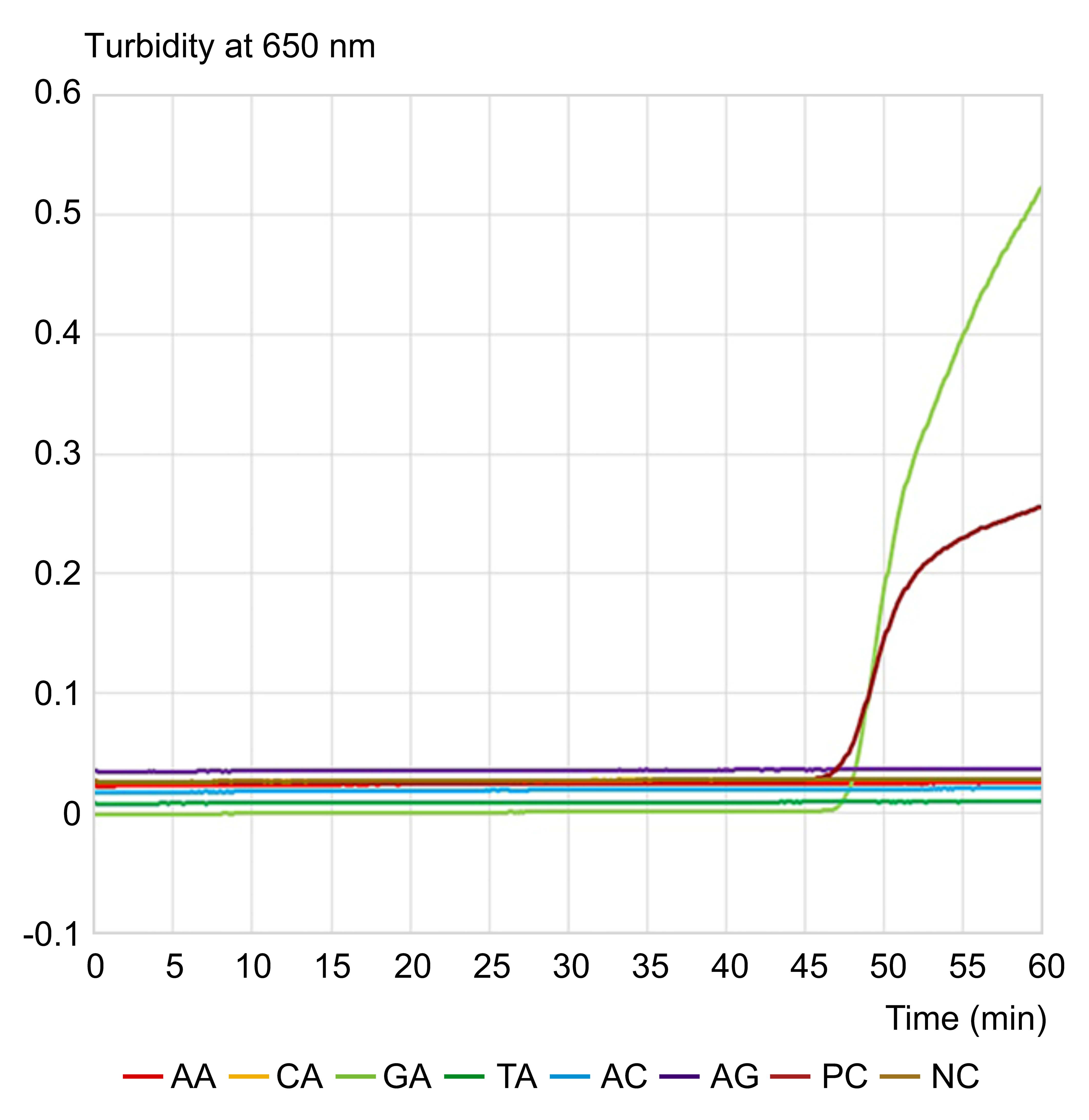


**Fig. S1.**


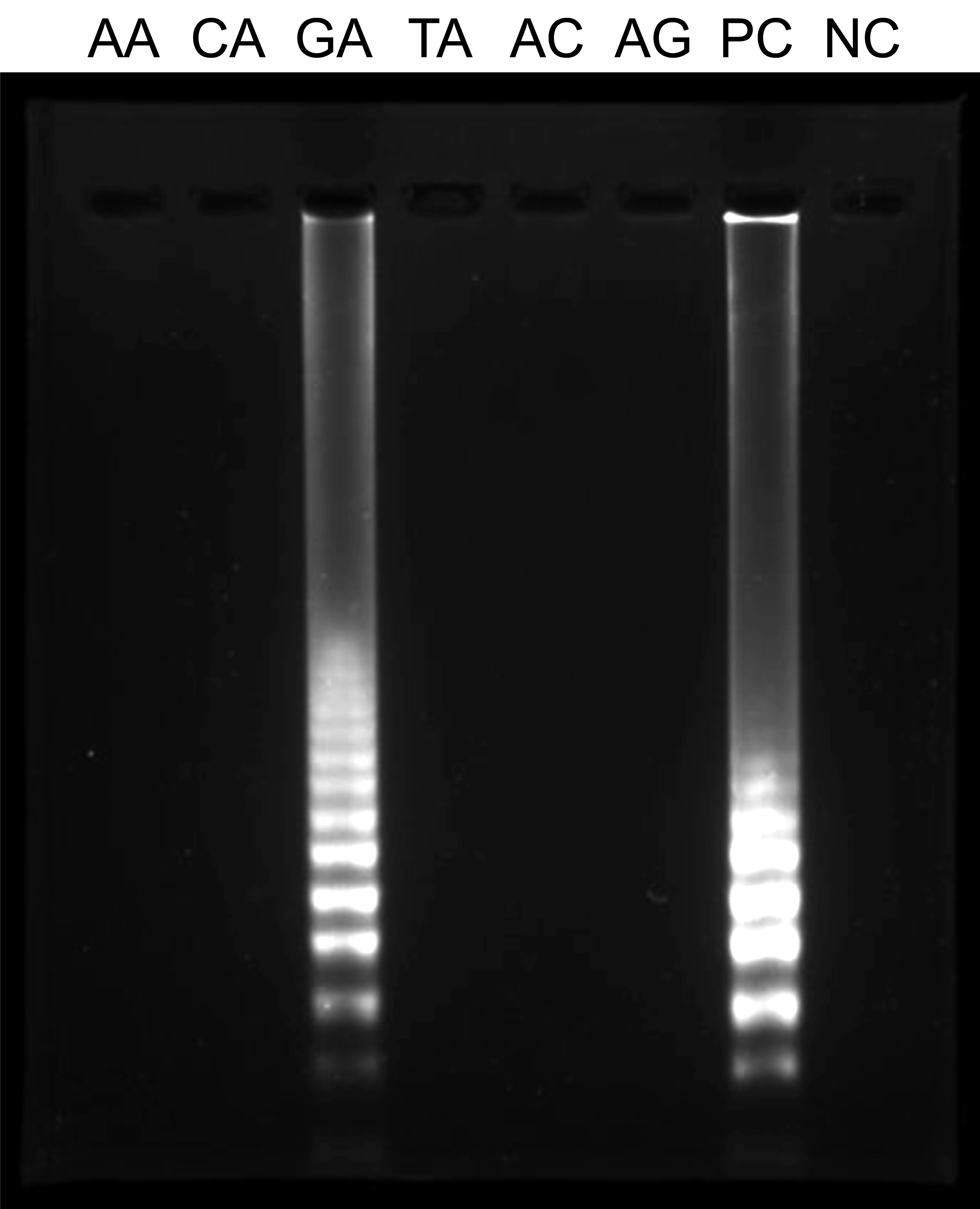


**Fig. S2.**
